# The type and scope of physiotherapy is under-utilised in Australian residential aged care facilities: a national, cross-sectional survey of physiotherapists

**DOI:** 10.1186/s12877-022-03248-4

**Published:** 2022-07-28

**Authors:** Lindsey Brett, Emre Ilhan

**Affiliations:** 1 Department of Health Sciences, Faculty of Medicine, Health and Human Sciences, Macquarie University, Sydney, NSW 2109 Australia; 2 Physiotherapy Department, Prince of Wales Hospital, 320-346 Barker Street, Randwick, NSW 2031 Australia

**Keywords:** Aged care, Nursing home, Physiotherapy, Physical therapy, Older adults

## Abstract

**Background:**

With an increasingly ageing population in Australia, more older adults who are frail are living in residential aged care facilities (RACFs). The aim of this study was to detail the type, scope, and funding of physiotherapy utilised in Australian RACFs.

**Methods:**

Registered physiotherapists (*n* = 219, 72% female, mean age (SD) = 38.6 (12.9) years) working in Australian RACFs participated in a nationwide, cross-sectional online survey. The survey was developed iteratively through a review of the literature and clinical guidelines, consensus of final survey items by an expert panel of five senior physiotherapists and aged care managers. Survey questions related to the characteristics of the physiotherapists (e.g., age, gender, employment status), characteristics of the RACFs (e.g., state, remoteness, sector), the type and scope of physiotherapy provided by respondents, and the availability of equipment and certain spaces (e.g., gyms) in the RACFs that respondents worked in. Survey responses were analysed and presented descriptively. Correlation using Spearman’s rho (ρ) and the associated 95% confidence intervals (CI) were used to determine whether the availability of equipment or space at the RACF was associated with the time dedicated to performing non-Aged Care Funding Instrument (ACFI) tasks.

**Results:**

Common reasons for physiotherapy referral were chronic pain management as per the ACFI framework (89.7%), falls (69.2%), and reduced mobility (35.9%). Rehabilitation or short-term restorative care was provided in only 22.2% of the facilities. The ACFI funded 91.4% of all participants, which limited physiotherapists to low-value chronic pain management including massage and electrical stimulation. Respondents spent 64.5% of their time on ACFI tasks, which equated to 19 h per week. More time was spent on non-ACFI tasks particularly when resistance bands (ρ = 0.28, 95%CI 0.14–0.41) and a dedicated therapy space or gym (ρ = 0.19, 95%CI 0.04–0.33) were available.

**Conclusions:**

The expertise of physiotherapists is currently being under-utilised in Australian RACFs, which may be related to the availability of public funding, equipment, and space for therapy. Therefore, public health policy should address the urgent need for high-value, evidence-based physiotherapy that supports the reablement and independence of older adults living in RACFs.

**Supplementary Information:**

The online version contains supplementary material available at 10.1186/s12877-022-03248-4.

## Background

Australian residential aged care facilities (RACFs) provide long-term accommodation and care to older adults who can no longer remain safely at home, and respite accommodation and care when older adults (or their carers) need a break from their usual living arrangements [1]. Allied health professionals (AHPs) can and should be utilized to help support the care needs, as well as restore and maintain the function of older adults living in RACFs [2–4]. Physiotherapists are the most common AHP in Australian RACFs; approximately 8% of physiotherapists predominantly work in RACFs [5]. Physiotherapists have the knowledge and skillset to autonomously support the varied, and often complex care needs of older adults in RACFs by addressing issues such as mobility impairments, pain, and incontinence [6]. Research has shown physiotherapy and exercise can positively impact physical performance, functional ability, falls risk and pain in older adults that live in RACFs [7–10]. These findings are supported by healthcare legislation and guidelines implemented to improve/maintain high standards of care in RACFS in countries such as Australia, the United Kingdom (UK), and the United States of America (USA), which recommend older adults living in RACFs are supported to utilize physiotherapy and exercise to improve and maintain function [3, 11, 12].

Funding of Australian RACFs is a combination of contributions by older adults living in such facilities and government subsidies. Older adults contribute 85% of a single age pension, as well as additional means-tested contributions, towards accommodation and care costs [13]. The Aged Care Funding Instrument (ACFI) is the main government subsidy in RACFs [14]. Physiotherapists are only funded by ACFI to provide either massage for a total of 20 min weekly (complex healthcare procedure 4a), or massage, electrotherapy, and other technical equipment for 20 min, 4 days per week solely for the purpose of pain management (complex healthcare procedure 4b) [15]. In October 2022, ACFI will be replaced by the Australian National Aged Care Classification (AN-ACC); aimed to disincentivise dependence of older adults in long-term care and promote a focus on restorative care and reablement [16]. Yet it does not provide funding for AHPs required to provide high-quality restorative and reablement care.

Similar to worldwide trends, the Australian population is ageing, resulting in older, frailer adults living in RACFs [17]. It is important they receive appropriate care from AHPs to maximize their function and quality of life. However, detailed information on the input of AHPs, such as physiotherapists, in Australian RACFs is widely unknown [18]. With the release of the Royal Commission into Quality and Safety of Aged Care final report and recommendations, and the government announcement to reform the Australian aged care sector, there is potential for improvement in access to and funding for AHPs. It is important to first understand the current system so both successful processes and shortcomings can be identified to guide future change. The aim of this study was to detail the current type, scope, and funding of physiotherapy utilized in Australian RACFs.

## Methods

### Design

Nationwide, cross-sectional online survey (Qualtrics platform) was held between September and December 2019. The first page of the survey included the participant information and consent form and required informed consent (tick-box response) prior to commencement of the survey. The study was approved by the Macquarie University Human Research Ethics Committee (reference: 5,201,955,849,573), registered on the Australian and New Zealand Clinical Trial Registry (reference: ACTRN12619001125112), and written in accordance with the Checklist for Reporting Of Survey Studies (CROSS) (Supplemental material Table 1) [19].

### Participants

Registered physiotherapists that mainly worked in Australian RACFs (at least 50% of their working week) at the time of the survey were included in this study. Physiotherapists with limited registration (overseas trained physiotherapists who practice under supervision) [20] or student status were excluded. The target sample size (*n* = 159) was based on the recommended 10% representation for survey studies [21]. At the time of the study development there were 1,588 physiotherapists that predominately worked in RACFs [5].

Potential participants were recruited indirectly using snowball sampling via email to aged care providers, social media, advertisements through relevant aged care and physiotherapy organisations, and promotion at related conferences and events. Invitations to participate, which included the anonymous survey link, were sent at the beginning, halfway point, and during the final week of the data collection period. The sampling methods selected prevented the ability to determine the recruitment rate as the denominator was unknown [22].

### Study measures

Based on clinical expertise of the research team, and review of previous research and clinical guidelines, a preliminary set of survey questions was developed. An expert panel (*n* = 5) that consisted of senior physiotherapists and RACF management reviewed and refined the survey until consensus was reached. The final survey (Supplemental material study survey) was pilot tested with physiotherapists that worked with older adults (*n* = 8) to check face validity and usability of the survey [23]. Other than for consent and screening, all other questions were voluntary. This was to encourage survey completion but may have resulted in missing data.

The survey asked questions related to participants’ demographics, characteristics of the RACFs in which they worked, the type and scope of physiotherapy services in Australian RACFs. The following data was collected and analysed as continuous variables: age, years worked as a registered physiotherapist, years worked in RACFs, average weekly working hours, number of RACFs worked at, number of providers worked for, percentage of older adults that received regular and ongoing physiotherapy, count of physiotherapists and other AHPs per RACF, ACFI- only AHPs per RACF, average time spent on specified type of sessions, duration of ACFI and non-ACFI referrals in a standard session, and percentage of time performing ACFI and non-ACFI tasks in a typical week. The following data were treated and analysed as categorical variables: gender, employment type, and presence of ACFI-only AHPs, RACF specialist care provisions, type of RACF, physiotherapist’s role, physiotherapy interventions, exercise types used, equipment and space available, equipment and space used (if available), outcome measures used, referral method, funding type, and reason/s for exclusion from physiotherapy. Data related to official job title, falls prevention strategies used, and respite services available at the RACF were also collected and analysed as categorical variables. Data related to the three most common conditions were collected and analysed as ordinal variables.

### Data analysis

Data was analysed using IBM SPSS Statistics for Macintosh, Version 27.0 (IBM Corp. in Armonk, NY). Descriptive statistics were used to determine the profile of responses to survey questions. Percentages represent the proportion of responses that have excluded missing values. Analysis of association between variables were performed using Spearman’s rho (ρ) to determine whether the availability of certain therapy equipment (e.g., walking aids) and/or space (e.g., a designated therapy room) was related to the percentage of total time in a typical working week that was dedicated to performing non-ACFI tasks. The 95% confidence intervals (95% CI) for the correlation coefficient were calculated using the Bonnett and Wright method. Where counts for some categories were less than 5, were reported as ‘<5’ to maintain the confidentiality of these unique responses.

## Results

### Participant and RACF characteristics

A total of 222 physiotherapists, who were mainly women (72%) with a mean (SD) age of 38.6 (12.9) years, participated in this study. Participant (2.7% missing data) and RACF (1.3% missing data) characteristics are detailed in Table 1 (and Supplemental material Table 2). Each state and territory of Australia was represented, mainly by participants that worked in major cities (67.6%) for an average (SD) of 30 (10) hours per week. The most common AHPs physiotherapists worked with in RACFs were podiatrists, other physiotherapists, and occupational therapists. On average, 53.1% (SD 22.9%) of older adults per RACF received ongoing physiotherapy.

**Table 1:** Characteristics of participants (*n* = 222) and the RACFs they worked in

**Physiotherapists**	
**Female**, n (%)	157 (71.7)
**Age**, mean (SD), years	38.6 (12.91)
**Average hours per week**, mean (SD)	30.1 (10)
**Employment status**,^a^n (%)	
Employed by RACF	61 (27.5)
Self-employed	20 (9.0)
Employed by external agency	145 (65.3)
**Number of RACFs worked at**, median (IQR)	1 (2)
**Years worked as physiotherapist**, median (IQR)	7 (19)
**Years worked in RACF**, median (IQR)	4 (6)
**RACFs**	
** Mean percentage of older adults receiving physiotherapy**, mean (SD)	53.1 (22.9)
**Remoteness,** n (%)^a^ *(3.2% missing data)*	
Major city	150 (69.8)
Inner regional	26 (12.1)
Outer regional	8 (3.7)
Very remote	< 5
Multiple regions	27 (12.6)
**State,** n (%)^a^ *(3.2% missing data)*	
New South Wales	79 (36.7)
Victoria	59 (27.4)
Queensland	26 (12.1)
Western Australia	21 (9.8)
South Australia	17 (7.9)
Tasmania	5 (2.3)
Other^b^	8 (3.7)
**Sector,** n (%)^a^ *(2.3% missing data)*	
Public	18 (8.3)
Private	122 (56.2)
Not-for-profit	116 (53.5)
Aboriginal cooperation	< 5
**Special services provided,** n (%)^a^ *(2.7% missing data)*	
Dementia	194 (89.8)
Respite	172 (79.6)
Palliative care	163 (75.5)
Rehabilitation/Short-term restorative care	48 (22.2)
Transition care	30 (13.9)
Mental health	29 (13.4)
Flexible care	26 (12.0)
Culture specific services/facilities	15 (6.9)
LGBTQI + support	14 (6.5)
Homeless support	7 (3.2)
Day hospital	< 5
Other^c^	< 5
None	6 (2.8)
**Type of respite service**, n (%) *(26.6% missing data)*	
Initial assessment	60 (36.8)
Falls review	38 (23.3)
Mobility assessments	37 (22.7)
Post-hospital discharge	< 5
Private physiotherapy	19 (11.7)
Equipment provision	11 (6.7)
Rehabilitation	5 (3.1)
Cardio-respiratory physiotherapy	< 5
Unspecified	10 (6.1)
Exercise prescription	32 (19.6)
Pain management	30 (18.4)

^a^ Categories are not mutually exclusive

^b^ Northern Territory and Australian Capital Territory

^c^ Other refers to categories with less than 5 counts which include disability care, hospital in the home, shared campus with local hospital

*IQR* Interquartile range; RACF Residential Aged Care Facility; *LGBTQI* + Lesbian, Gay, Bisexual, Transgender, Queer, and Intersex;

*AHPs* Allied Health Professionals; *SD* standard deviation

The median number of additional services that RACFs provided was 3 (interquartile range = 2, range = 0–8); the most common being dementia care (87.4%) and the least day hospital care (1.8%). Rehabilitation or short-term restorative care was most often provided by not-for-profit RACFs (62.5%). Only 5.4% of physiotherapists worked in RACFs that provided minimal to no physiotherapy services to older adults in respite, whereas 6.1% of participants reported that older adults received the same type of services regardless of respite/permanent status. The most common intervention provided by physiotherapists to older adults in respite was an initial assessment (36.8%).

### Type and scope of physiotherapy

Almost a third of participants (30.8%) reported that no older adults were excluded from physiotherapy in the RACFs they worked in, of those that did, funding was a commonly cited reason: either due to maximum ACFI claim being reached (41.3%), or older adults lacked funding (40.1%).

**Fig. 1:**
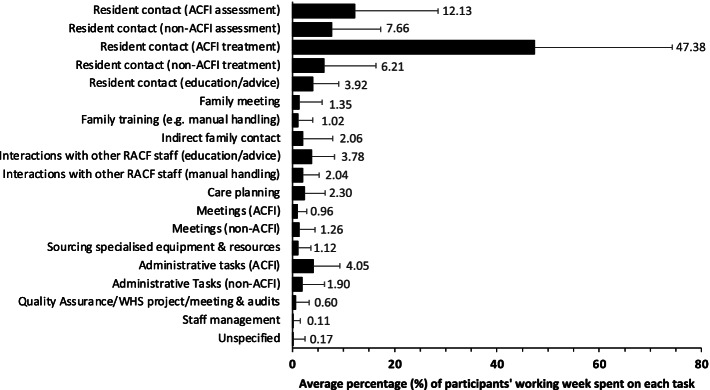
Average percentage (%) of participants’ typical working week spent on completing different physiotherapy activities (x-axis) (error bars represent standard deviations in percentage)

Physiotherapy referrals were either from members of the multidisciplinary team, older adults, and their families, or incorporated into routine care (e.g., on admission). The most common referral source were nurses (87.4%), and the least common appointed external personnel (e.g., public trustee, person of authority, Department of Veteran’s Affairs [DVA] representative, or National Disability Insurance Scheme [NDIS] coordinator) (1.1%). The type and scope of physiotherapy in Australian RACFs are detailed in Table 2 (and Supplemental material Table 3). The proportion of time spent on different physiotherapy activities per average week are displayed in Fig. 1.

**Table 2:** Type and scope of physiotherapy in Australian RACFs

Variable	n (%)
**Most common reason for physiotherapy referral** *(12.2% missing data)*	
Management of chronic pain as per ACFI framework	175 (89.7)
Management of chronic pain – non-ACFI	10 (5.1)
Falls	135 (69.2)
Reduced mobility	70 (35.9)
Decline in function/deconditioning	53 (27.2)
New admission	37 (19.0)
Post-hospital discharge	7 (3.6)
Regular/routine review	75 (38.5)
Dementia	14 (7.2)
Other^a^	5 (2.6)
**Source of physiotherapy referral** *(21.2% missing data)*	
General practitioner	114 (65.1)
Nurse	153 (87.4)
Resident	79 (45.1)
Family	79 (45.1)
Routine for new admission	144 (82.3)
Routine for scheduled review	129 (73.7)
Blanket referral for specific incidents/issues	116 (66.3)
Other^b^	< 5
**Physiotherapy role** *(8.6% missing data)*	
ACFI Chronic pain management	193 (93.6)
***ACFI Chronic pain management interventions**** (4.9% missing data)*	
*Massage for complex healthcare procedure 4a*	107 (55.4)
*Massage for complex healthcare procedure 4b*	180 (93.3)
*Electrotherapy or other technical equipment for complex healthcare procedure 4b*	69 (35.8)
Non-ACFI Chronic pain management	69 (34.0)
Falls prevention and treatment	156 (76.8)
***Falls prevention and treatment interventions**** (13.5% missing data)*	
*Exercise*	125 (92.6)
*Education/advice*	53 (39.3)
*Harm and risk minimization*	65 (48.1)
*Equipment and apparel*	51 (37.8)
*Personal processes*	11 (8.1)
*Referral to and collaboration with other health care professionals*	11 (8.1)
*Reviews/audits*	27 (20.0)
*Environmental modifications*	22 (16.3)
*Other medical and pain management*	7 (5.2)
Mobility and functional maintenance	153 (75.4)
Rehabilitation/short-term restorative care	85 (41.9)
Staff training and consultation	123 (60.6)
Equipment recommendations and provision	5 (2.3)
Other^c^	21 (10.3)
**Validated outcome measures utilized** (*n* = 30) *(21.2% missing data)*	
Pain (*n* = 5)	162 (92.6)
Balance and falls risk (*n* = 6)	121 (69.1)
Functional (*n* = 3)	104 (59.4)
Mobility (*n* = 6)	62 (35.4)
Multi-faceted: mobility and balance or function (*n* = 3)	21 (12.0)
Functional lower limb strength (*n* = 2)	74 (42.3)
Overall health (*n* = 3)	9 (5.1)
Other^d^ (*n* = 2)	< 5
**Reason older adult excluded from physiotherapy** *(22.5% missing data)*	
None excluded	53 (30.8)
Maximum ACFI claim reached	71 (41.3)
Lack of funding	69 (40.1)
Respite status	62 (36.0)
Non-compliance of older adult and/or family with physiotherapy	22 (12.8)
Cognitive impairment	19 (11.0)
Other^e^	10 (5.8)
**Duration (minutes) of physiotherapy sessions**, mean (SD) *(19.8% missing data)*	
New admission assessment	35.3 (29.8)
Discharge session	3.9 (9.8)
Regular scheduled review	18.5 (15.2)
Unscheduled review	16.7 (15.9)
Falls review	18.9 (13.5)
Resident and family liaising	11.4 (10.9)

*ACFI* Aged Care Funding Instrument, *RACF* Residential Aged Care Facility, *SD* standard deviation

^a^ Other refers to categories with less than 5 counts which include stroke; Parkinson’s disease; oedema; and change in neurological function

^b^ Other refers to categories with less than 5 counts which include appointed external personnel and ACFI coordinator

^c^ Other refers to categories with less than 5 counts which include respiratory physiotherapy; quality improvement projects; resident and family education and training; cardiac physiotherapy; staff management; reviews and case conferences; Work Health and Safety, return-to-work, and manual handling; pressure area care; exercise prescription (individual or group)

^d^ Other refers to categories with less than 5 counts which include nine-hole peg test and Goal Attainment Scale (GAS)

^e^ Other refers to categories with less than 5 counts which include behavioural issues; insufficient number of physiotherapists for caseload; insufficient time for caseload; not indicated

The three most common reasons for physiotherapy referrals were management of chronic pain as per the ACFI framework (89.7%), falls (69.2%), and reduced mobility (35.9%). Similarly, the three most common physiotherapy roles were chronic pain management under the ACFI framework (93.6%), falls management strategies (76.8%), and interventions to address reduced mobility and function (75.4%). There were a wide range of outcome measures used by physiotherapists in RACFs (*n* = 30), which were categorized by the primary focus of the measure. The three most common types of outcome measure were pain (92.6%), balance and falls risk (69.1%), and functional (59.4%), which align with the most common reason for referral to physiotherapy, and the most common physiotherapy roles.

## Funding

Source of funding was reported by 175 participants (21.2% missing data). Physiotherapy was reported to be funded predominately by ACFI (91.4%). The next most common sources relied on funds directly from the RACF (57.1%) or the older adult (28.0%). Whereas other government and private insurance funding sources were utilized at much lower rates: Medicare (17.7%), DVA (19.4%), private health insurance (14.9%), and others which were reported by less than five participants each (NDIS, transitional care package, religious organization and a combination of State and Commonwealth sources).

### ACFI vs non-ACFI physiotherapy

Data related to duration spent on ACFI and non-ACFI physiotherapy was available for 80.1% of participants. Participants spent, on average, 64.5% (SD 25.2) of their time completing ACFI tasks in a typical work week, which equated to an average of 19 h (9.7) per week. The mean (SD) duration spent on administering ACFI-funded sessions was 28 (29) minutes for assessments, and 24 (51) minutes for treatments. This contrasts with non-ACFI sessions which had a mean (SD) duration of 17 (18) minutes for assessments, and 13 (11) minutes for treatments. The duration of time a physiotherapist would see an older adult for a referral also varied based on the referral type; input for ACFI referrals were more often 12 weeks or longer (79.3%) with less than 9% of reviews being single reviews or being less than 12 weeks. Input for non-ACFI referrals lasted four weeks or less (33.9%), consisted of a single intervention only (24.0%), 4 to 12 weeks long (18.2%), 12 weeks or longer (13.2%), or not stated (10.7%).

**Fig. 2:**
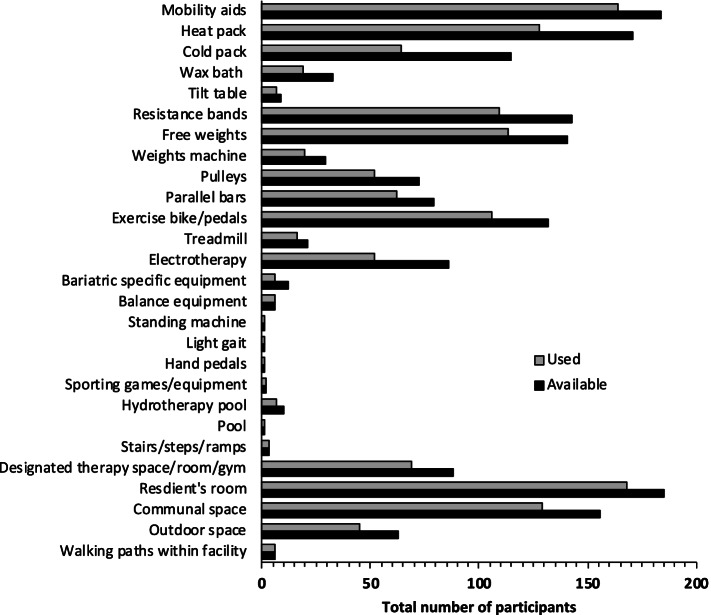
Total number of participants who indicated that the equipment and facilities were used versus available

A correlation analysis (*Spearman’s* ρ) was performed to determine whether the availability of the equipment and facilities considered in this study (outlined in Fig. 2) correlated with the percentage of total time in a typical working week dedicated to non-ACFI tasks (Table 3). The availability of resistance bands (ρ = 0.28, 95% CI 0.14 to 0.41), free weights (ρ = 0.17, 95% CI 0.02 to 0.31), exercise bikes/pedals (ρ = 0.23, 95% CI 0.08 to 0.36), treadmill (ρ = 0.15, 95% CI 0.01 to 0.30), and having a designated therapy space/gym (ρ = 0.19, 95% CI 0.04 to 0.32) was associated with more time spent on non-ACFI tasks.

**Table 3:** Correlation between equipment and facility availability with percentage (%) of total time spent on non-ACFI tasks in a typical week

**Equipment available**	**ρ (95% CI)**
Mobility aid	-0.01 (-0.16, 0.13)
Heat pack	0.09 (-0.05, 0.23)
Cold pack	0.07 (-0.08, 0.21)
Wax bath	0.11 (-0.03, 0.26)
Tilt table	0.14 (-0.00, 0.28)
Resistance band	**0.28 (0.14, 0.41)**
Free weights	**0.17 (0.02, 0.31)**
Weights machine	0.14 (-0.01, 0.28)
Pulleys	0.11 (-0.04, 0.25)
Parallel bars	0.14 (-0.01, 0.28)
Exercise bike/pedals	**0.23 (0.08, 0.36)**
Treadmill	**0.15 (0.01, 0.29)**
Electrotherapy	0.00 (-0.14, 0.15)
Bariatric specific equipment	0.11 (-0.04, 0.25)
Balance equipment	-0.03 (-0.17, 0.12)
Standing machine	-0.10 (-0.24, 0.05)
Light gait	0.07 (-0.08, 0.21)
Hand pedals	-0.08 (-0.22, 0.07)
Sporting games/equipment	0.05 (-0.10, 0.19)
**Facility available**	
Hydrotherapy pool	0.11 (-0.04, 0.25)
Pool	0.12 (-0.02, 0.27)
Stairs/steps/ramp	0.07 (-0.08, 0.21)
Designated therapy space/room/gym	**0.19 (0.04, 0.32)**
Resident’s room	-0.04 (-0.18, 0.11)
Communal space	0.15 (0.00, 0.29)
Outdoor space	0.13 (-0.02, 0.27)
Walking paths within facility	0.01 (-0.14, 0.15)

*ρ* Spearman’s rho, *ACFI* Aged Care Funding Instrument

Bolded correlation coefficients were significant at a *p* value < 0.05

## Discussion

The study provides the first detailed snapshot of the physiotherapy workforce in Australian RACFs, including type, scope, and funding. While this study had a representative sample of physiotherapists according to physiotherapy workforce data collected by the Australian Health Practitioner Regulation Agency (AHPRA) in 2019 [5], there were 6.4% more women, 5.5% more physiotherapists from New South Wales, and 8.1% less physiotherapists from Queensland compared to national data.

The proportion of older adults in Australian RACFs that received physiotherapy (53.12%) as reported in this study was similar to studies from the UK (10.4–76%) [24–26], which may be associated with the similarities between the healthcare systems. Data from this study show a greater proportion of older adults receive physiotherapy in comparison to Canada (11%), Italy (12%), Denmark (19%), Japan (20%), Iceland (32%), and USA (14–43.9%) [27–29], but much lower compared to the Netherlands (67.3–99%) [30, 31]. This may be attributed to the strong rehabilitation approach and consideration of AHPs as essential in Dutch RACFs [30].

Older adults living in RACFs are five times more likely to fall than their community-dwelling counterparts [32], yet there is a lack of high-quality research on falls prevention and management in RACFs [32, 33], and absence of a ‘gold standard’ option for assessment tools and treatment interventions. A report from the Royal Commission into Aged Care Quality and Safety found the most common medical and healthcare concerns of older adults in RACFs was falls and falls prevention [34], but there is no funding that specifically addresses this issue in Australian RACFs.

Despite the lack of high-quality research and funding, this study found that falls management was the second most reported reason for physiotherapy referral (69.2%) and activity (76.8%), and balance and falls outcome measures were the second most utilized outcome measures (69.1%). Physiotherapists used up to seven of the nine reported falls prevention and management interventions, including exercise (92.6%), equipment and apparel provision (e.g., mobility aids and hip protectors) (37.8%), and collaboration with other healthcare professionals (8.1%). Such interventions aligns with best-practice guidelines for reducing falls in Australian RACFs: A multifactorial approach that includes vitamin D and calcium supplementation, medication review, exercise, and hip protectors) [32, 33]. Fall prevention is one of the few issues that has guidelines specific to the RACF population. However, they are based on very low to moderate -quality evidence. Limited research relevant to the unique needs of older adults living in RACFs makes it difficult to determining if current guidelines and practice are in fact best practice [35].

The most cited reason for physiotherapy (93.6%) in this study was management of chronic pain as per ACFI rules. Given chronic pain affects up to 82.9% of older adults living in RACFs [36], funding for chronic pain in RACFs is justifiable; however, ACFI only funds a narrow set of treatments for chronic pain, all of which have limited evidence supporting their use in older adults. This highlights a funding model that is neither person-centred nor based on the best available evidence [37, 38]. A recent qualitative study echoes the position that organisational factors such as policies, time availabilities, and capacity for multi-disciplinary input influence the extent to which physiotherapists are involved in the management of older adults in aged care [39]. Current best practice for physiotherapy-specific interventions for chronic pain in RACFs recommend a multi-modal approach including resistance, aerobic, and stretching exercises, advice and education on pain, and training of staff around manual handling, in addition to passive modalities such as heat and TENS [40]. Despite the availability of evidence to improve pain management among older adults in RACFs, the AN-ACC [16], the new funding model to replace ACFI in October 2022, and an increased government budget in light of the Royal Commission into Aged Care Quality and Safety have not included these recommendations or specific AHP funding. This may have detrimental effects on the wellbeing of older adults in Australian RACFs, which may also include limiting residents’ access to high-quality care by physiotherapists.

Along with equitable access to AHPs and evidence-based care, the availability and use of suitable equipment and facilities are also important considerations when evaluating a RACF’s capacity to re-enable and rehabilitate older adults. This study found that the availability of certain exercise equipment including resistance bands, free weights, exercise bikes/pedals, treadmills, and a designated therapy space were associated with greater time spent on non-ACFI tasks per week. This highlights the importance of equipment and space availability to enable physiotherapists to provide high-quality care to older adults in RACFs. Physical activity and exercise are encouraged throughout life and associated with a number of health benefits [41]. Yet when older adults move to a RACF the emphasis on physical activity and exercise may be removed. Proprietors of RACFs should, in part, take on the responsibility to provide suitable equipment and environments to promote a reablement model of care and the utilization of AHPs such as physiotherapists to support older adults living in RACFs. Research has shown the implementation of an enriched environment, in addition to therapy, and evidence-based care can potentially increase opportunities for physical, cognitive, and social activity [42]. All of which could help to maintain function, independence, and quality of life for older adults living in RACFs.

Respite was the second most common special service provided in Australian RACFs (77.5%); however, physiotherapists’ input was very basic consisting of initial assessments and post-falls reviews, with only 2.3% providing any rehabilitation to older adults in respite. Over a third of physiotherapists in this study reported that respite was a reason for exclusion from physiotherapy. Consideration of the services required to support older adults in RACF respite is important. Older adults who are admitted to respite from the acute setting may be misinformed about the access to services in respite, such as physiotherapy and other allied health services. Along with frustration, the lack of AHP input can often lead to deterioration in functional ability of the older adult, which can have a detrimental impact on where they transfer to following respite; in 2008–09, 40% of older adults admitted to a RACF for respite had transferred to permanent care, 15% were discharged back to hospital, and 9% had died within 12 weeks of their respite admission [43]. Ideally, a change to a reablement approach to respite care and future funding should allow older adults the opportunity to maximize their potential to return home through greater input of AHPs. This could result in greater utilization of respite services, reducing the length of stay in hospital as well as associated costs. Potential acute care cost savings could then be used to support an improved respite service in RACFs that utilizes AHPs.

## Strengths and limitations

The ongoing health and wellbeing of older adults in RACFs is a major public health concern. This study provides valuable data about physiotherapy in Australian RACFs that was previously unknown. It highlights current scope as well as potential limitations which need to be considered to ensure physiotherapists are supported by public health measures and policies to work autonomously and are funded to provide equitable and effective care to all older adults in RACFs. However, this study has some limitations. The survey was voluntary and due to the methods used (snowball sampling, anonymous link to access survey, multiple means of advertising the survey) it was not possible to determine the response rate, it may have only been completed by proactive physiotherapists, and thus not representative of all RACF physiotherapists in Australia. However, the characteristics of the physiotherapists in this study were similar to that collected by AHPRA, which suggests that a representative group of physiotherapists was sampled. Physiotherapists were asked to recall information about the RACFs they worked in, of which they may not have had a full understanding and limited the available data associated with RACFs characteristics. Future research could address this limitation by including RACF managers. Another limitation associated with a survey design is the risk of missing responses. The survey had 1.3–26.6% missing data across 16 of the 23 variables; however, only eight variables had more than 5% data missing.

## Conclusions

This study has highlighted the need for further high-quality research specific to older adults living in RACFs, including subpopulations such as those living with a cognitive impairment or frailty, to support implementation of evidence-based practice. Public health policy makers should consider the need for appropriate government funding to promote equitable access to evidence-based physiotherapy that addresses major health issues in RACFs, such as falls prevention and chronic pain management. At a local level, to optimize the funding of physiotherapists and other AHPs, RACF organizations should ensure their facilities offer suitable equipment and space to utilize the skillsets and promote a reablement model of care. Finally, from an individual level, physiotherapists should take responsibility to ensure they are up to date with evidence-based recommendations through professional development opportunities. They can then use their expertise to educate RACF staff, older adults and their families on the benefits associated with physiotherapy, and advocate for better care of older adults at a local level.

This study detailed how physiotherapists are currently being utilized in Australian RACFs. Physiotherapists working in aged care have a large skillset which could be better utilized to benefit older Australians. This may, for example, include a greater utilization of exercise to support reablement and independence. Further research is also needed to identify the most effective approaches to assessment and treatment of older adults that live in RACFs. A consideration of the data presented in this study when developing future funding and care models may improve equitable access to physiotherapy for older adults living in RACFs and support a better aged care sector in general.

## Supplementary Information

**Additional file 1:** **Supplemental material.** Study survey.

**Additional file 2:** **Supporting Information Table 1.** Checklist for Reporting Of Survey Studies (CROSS).

**Additional file 3:** **Supporting Information Table 2 Extension of Table 1.** Allied health professionals in RACFs.

**Additional file 4:** **Supporting Information Table 3 Extension of Table 2.** Detailed interventions and outcome measures reported.

## Data Availability

All data generated or analysed during this study are included in this published article and its supplementary information files.

## Abbreviations

ACFI: Aged care funding instrument
AHP: Allied health professional
AN-ACC: Australian national aged care classification
DVA: Department of Veteran’s Affairs
NDIS: National Disability Insurance Scheme
RACF: Residential aged care facility
