# Supplementary material for: The type and scope of physiotherapy is under-utilised in Australian residential aged care facilities: a national, cross-sectional survey of physiotherapists
Source: BMC Geriatr. 2022 Jul 28;22:625. doi: 10.1186/s12877-022-03248-4 (PMC9331124; doi:10.1186/s12877-022-03248-4)
Supplement: Supplementary file 1 — Additional file 1: Supplemental material. Study survey. [file 12877_2022_3248_MOESM1_ESM.pdf]

Tools Saved at 7:37 PM Draft

Preview Publish

Physiotherapy services utilised in Australian RACFs

iQ Score: Fair

Information & Consent Form

Q1

Examining physiotherapy services utilised by older Australians living in residential aged care facilities: clinician survey

Thank you for showing interest in this research project. This page provides detailed information about this study and the associated survey. Please take the time to read this information carefully and only proceed to the survey if you consent to being involved. Moving to the next part of this survey will serve as your provision of consent.

Please click the ">>" button below to move to the next page. Should you wish to go back a page at any stage you can use the "<<" button.

What is the study about?
You are invited to participate in a study which is exploring the current status of physiotherapy services within the Residential Aged Care Facility (RACF) sector. With substantially increasing numbers of Australians aged 65 years and older, there is an increased demand on RACFs. Physiotherapists working in this sector have the skills and knowledge to assist with mobility and movement related dysfunctions, rehabilitation, pain management, fall and injury risk minimisation, individualised exercise prescription, incontinence management, and manual handling education.

Despite Physiotherapists being well placed to assist in the care needs of older Australians, current government schemes only fund allied health services related to pain management. This includes massage, electrotherapy and other technical equipment which are not supported by strong literature as effective treatment for pain management.

This study hopes to survey clinicians and gain insight related to current physiotherapy practice in the RACF space. This project has the potential to impact on policy and practice to ensure effective and equitable physiotherapy is available to all older adults living in Australian RACFs.

Who is carrying out the study?
The study is being conducted by [redacted] from the [redacted]
Please note there will also be a research assistant and students [redacted].

What does the study involve?
If you agree to participate, you will be asked to complete a short survey. In the survey, we will ask you to provide some basic non-identifiable information about yourself, your clinical experience within the RACF sector and current work practice. These questions will take approximately 15-25 minutes to complete and no further involvement is required as part of this project.

Will my responses remain confidential?
All aspects of the study, including results, will be strictly confidential and only the

[illegible] researchers listed on this study will have access to the information provided. The survey has been developed to ensure that personally identifiable information is not collected. This is to ensure participants are willing to provide honest feedback without concern of anonymity being compromised. All results from this study will be consolidated and presented as group results, no individual data will be reported.

Is participation in this study voluntary?
Participation in this study is entirely voluntary. You are not obligated to participate, and if you decide to participate, you are free to withdraw (abort the survey) at any time without having to give a reason and without consequence. Please note that due to the anonymous nature of data collected in this survey, the research team cannot identify your individual data once you have submitted your responses. You can review your data by toggling through the 'back' button of the survey, but once you hit 'submit' we unfortunately cannot identify and withdraw your responses.

Are there any benefits and/or risks to participating in this survey?
There is no individual benefit associated with involvement in this study. However, it is anticipated that this project will provide detailed information regarding the current scope of physiotherapy in RACF. This information hopes to further educate the wider physiotherapy community and demonstrate how the professions knowledge and skillset can be utilised beyond current practice.

Aside from the time-burden of completing the survey (approximately 15-25 minutes), there is no anticipated burden associated with involvement in this study.

Can I contact the researchers?
If you have any questions related to the survey, please contact a member of the research team:
[redacted]

What if I have concerns or complaints?
The ethical aspects of this study have been approved by the [redacted] Human Research Ethics Committee. If you have any complaints or reservations about any ethical aspect of your participation in this research, you may contact the Committee through the Director, Research Ethics & Integrity [redacted]. Any complaint you make will be treated in confidence and investigated, and you will be informed of the outcome.

How to provide consent?
After reading the above document, if you are willing to proceed and consent to participate in the survey – please click the ">>" button below to complete the survey. If at any stage you would like to withdraw from the project, please abort the survey. Once again, many thanks for your time and interest in this project.

Import from library Add new question

Add Block

Inclusion criteria

Q33 *

Skip to

End of Survey if No Is Selected

Are you a qualified physiotherapist that currently practices in Australia and has full registration with AHPRA?

- ◯ Yes
- ◯ No

Q32 *

Skip to

End of Survey if No Is Selected

Regardless of if you work full time, part time or casual, do you spend at least 50% of your working week in Residential Aged Care Facilities (RACFs)?

- ◯ Yes
- ◯ No

Import from library | Add new question

Add Block

Demographic Questions

Q2 iQ *

What year was you born?
(please write year in full, e.g. 1982)

Q3

Please indicate your gender

- ◯ Male
- ◯ Female
- ◯ Prefer to not disclose
- ◯ Other

Page Break

Q7

How many years have you worked as a qualified physiotherapist (across all settings)?

Q8

How many years have you worked in RACFs?

Q4

What is your official job title?

Import from library Add new question

Add Block

Work Force and RACF characteristics Questions

Q6

On average, how many hours do you spend working in RACFs per week?

Q15

Please indicate what type of employment is most descriptive of your role in RACF(s)
(tick as many boxes as relevant)

- ☐ Employed by RACF
- ☐ Contracted - Self employed
- ☐ Contracted - Through external company/agency
- ☐ Other:

Q41

How many RACFs do you work at?

	0	1	2	3	4	5	6	7	8	9	10

Q40

Please provide the postcode for the RACF(s) you work in

	Postcode
RACF 1	
RACF 2	
RACF 3	
RACF 4	
RACF 5	
RACF 6	
RACF 7	
RACF 8	
RACF 9	
RACF 10	

Q36

Display this question

If Please provide the postcode for the RACF(s) you work in Text Response Is Not Empty

How many different residential aged care groups/organisations do you work for/with?

	0	1	2	3	4	5	6	7	8	9	10
Number of groups/organisations											

Page Break

Q10

Please indicate what type of RACF(s) you work in?
(tick as many options as relevant)

- ☐ Private sector
- ☐ Not-for-profit
- ☐ Public sector/State Government
- ☐ Other:

Q13

Please indicate the type of specialist care provided in the RACF(s) at which you work?
(tick as many options as are relevant)

- ☐ Rehabilitation/Short term restorative care
- ☐ Day Hospital
- ☐ Transition care
- ☐ Respite
- ☐ Flexible care
- ☐ Palliative care
- ☐ Dementia
- ☐ Mental health
- ☐ LGBTI support services
- ☐ Homeless support services
- ☐ Cultural specific services/facility, which culture(s)? ______
- ☐ Other: ______
- ☐ None

Page Break

Q22

Please indicate how many of the following allied health professionals (including yourself) work in the same RACF(s) in which you currently work (e.g. Type: Occupational Therapist; RACF 1: 4)
(if none of a particular profession work in the same RACF as you leave the box blank)

	RACF 1	RACF 2	RACF 3	RACF 4	RACF 5	RACF 6	RACF 7	RACF 8	RACF 9	RACF 10
Physiotherapist										
Occupational Therapist										
Podiatrist										
Chiropractor										
Osteopath										
Exercise Physiologist										
Diversional Therapist										
Dietician										
Speech & Language Therapist										
Palliative care consultant/nurse										
Physiotherapy Assistant										
Occupational Therapy Assistant										
Allied Health Assistant (AHA) (please state specialty)										
Other (please state specialty)										
Other (please state specialty)										

Q35

Do any allied health professionals (including yourself if applicable) at any of the RACFs you work at only complete ACFI assessments and/or treatments?

- ○ Yes
- ○ No
- ○ Unsure

Q41

iQ

Display this question

If Do any allied health professionals (including yourself if applicable) at any of the RACFs you wor... Yes Is Selected

Please indicate how many of the following allied health professionals (including yourself if applicable) only complete ACFI assessments and/or treatments for each RACF you work at (e.g. Type: Occupational Therapist; RACF 1: 2)
(if none of a particular profession work in the same RACF as you leave the box blank)

	RACF 1	RACF 2	RACF 3	RACF 4	RACF 5	RACF 6	RACF 7	RACF 8	RACF 9	RACF 10
Physiotherapist										
Occupational Therapist										
Podiatrist										
Chiropractor										
Osteopath										

Import from library

Add new question

Add Block

Physiotherapy Treatment Questions

Q11

Approximately what percentage of residents at your facility receive regular, ongoing physiotherapy?
(if you work at more than one RACF please provide percentage for each site)

	0	10	20	30	40	50	60	70	80	90	100
Percentage (%) at RACF 1											
Percentage (%) at RACF 2											
Percentage (%) at RACF 3											
Percentage (%) at RACF 4											
Percentage (%) at RACF 5											
Percentage (%) at RACF 6											
Percentage (%) at RACF 7											
Percentage (%) at RACF 8											
Percentage (%) at RACF 9											
Percentage (%) at RACF 10											

Q21

Under which broad categories does your role fall under?

- ☐ Pain Management (ACFI)
- ☐ Pain Management (non-ACFI)
- ☐ Falls prevention and treatment
- ☐ Mobility and functional maintenance
- ☐ Rehabilitation/short term restorative care
- ☐ Staff consultation and training (e.g. manual handling, task training)
- ☐ Other: ______

Q36

Display this question

If Under which broad categories does your role fall under? Falls prevention and treatment Is Selected

Please specify falls prevention strategies you use (e.g. type of exercises, specific programs and equipment)

Page Break

Q25

Considering all the RACFs you work at, please indicate the most common (top 3) conditions and issues you treat in your role? Please rate from 1 to 3 with the most commonly treated = 1.

- Falls
- Reduced Mobility
- Decline in Function/Deconditioning
- Pain (ACFI)
- Pain (non-ACFI)
- New Admission Assessment
- Post-Hospital Discharge
- Regular/general reviews, how often (e.g. annual, 4 monthly)?
- Stroke
- Parkinson's Disease
- Dementia
- Other:
- Other:

Page Break

Q14

What type of physiotherapy-based interventions do you utilise in your workplace? (please tick as many options as are relevant, and add any other interventions used as required)

- ☐ Exercise (one to one)
- ☐ Exercise (group - conducted by physiotherapist)
- ☐ Exercise (group - supervision of/in conjunction with other RACF staff/AHP/AHA)
- ☐ Exercise (supervision/review of self-managed program by resident/family/RACF staff)
- ☐ Massage provided for CHC 4a in ACFI
- ☐ Massage provided for CHC 4b in ACFI
- ☐ Massage - other
- ☐ Heat and/or Cryotherapy
- ☐ Dry needling or Acupuncture
- ☐ Education (staff)
- ☐ Education (resident and family)
- ☐ Electrotherapy and other technical equipment provided for CHC 4b in ACFI (please specify equipment used) ________
- ☐ Electrotherapy and other technical equipment - other (please specify equipment used) ________
- ☐ Manual therapy (please specify techniques and equipment used) ________
- ☐ Chest physiotherapy (please specify techniques and equipment used) ________
- ☐ Equipment prescription (please specify type of equipment) ________
- ☐ Other: ________

Q31

Display this question

If What type of physiotherapy-based interventions do you utilise in your workplace? (please tick as... Exercise (group - conducted by physiotherapist) Is Selected

Or What type of physiotherapy-based interventions do you utilise in your workplace? (please tick as... Exercise (group - supervision of/in conjunction with other RACF staff/AHP/AHA) Is Selected

Or What type of physiotherapy-based interventions do you utilise in your workplace? (please tick as... Exercise (supervision/review of self-managed program by resident/family/RACF staff) Is Selected

Or What type of physiotherapy-based interventions do you utilise in your workplace? (please tick as... Exercise (one to one) Is Selected

If you utilise exercise at the RACF(s) you work at, what type of exercise do you use (please tick as many options as are relevant, and add any other types used as required)?

- ☐ Balance
- ☐ Strength
- ☐ Maintenance of range of movement/motion
- ☐ Endurance
- ☐ Walking and gait re-education
- ☐ Functional
- ☐ Tai Chi
- ☐ Dance
- ☐ Pilates
- ☐ Hydrotherapy
- ☐ Vestibular (e.g. BPPV)
- ☐ Pelvic floor muscle training
- ☐ Other: ____________

Page Break

Q16

What type of equipment is **available** to you, and what type of equipment do you **use** whilst working at your RACF(s)?
(please tick as many options as are relevant, and add any other forms of equipment not listed)

	Available	Use
Walking Aids	☐	☐
Heat pack	☐	☐
Cold pack	☐	☐
Wax bath	☐	☐
Tilt Table	☐	☐
Resistance Bands	☐	☐
Free weights	☐	☐
Weights Machine	☐	☐
Pulleys	☐	☐
Parallel Bars	☐	☐
Exercise Bike/pedals	☐	☐
Treadmill	☐	☐
Hydrotherapy pool	☐	☐
Electrotherapy (please specify)	☐	☐
Bariatric specific (please specify)	☐	☐
Other:	☐	☐
Other:	☐	☐

Q18

Please indicate what treatment spaces are available and used whilst working at your RACF(s)?

	Available	Used
Designated physiotherapy/therapy space or room	☐	☐
Resident's room	☐	☐
Communal space/room (e.g. lounge)	☐	☐
Outdoor space catering to treatment	☐	☐
Other:	☐	☐
Other:	☐	☐

Page Break

Q19

What does a typical work week entail? Please provide a percentage of time to indicate the time spent on each activity (ensuring it adds to 100%)

0 10 20 30 40 50 60 70 80 90 100

Resident contact - ACFI assessment

Resident contact - non-ACFI assessment

Resident contact - ACFI treatment

Resident contact – non-ACFI treatment

Resident contact - education/advice

Family meeting

Family training (e.g. manual handling, task training)

Indirect family contact (please specify by which means, e.g. telephone, face to face, email) ____

Interactions with other RACF staff - education/advice

Interactions with other RACF staff - manual handling, task training

Care planning

Meetings - ACFI

Meetings - non-ACFI (please specify, e.g. falls and incidents committee, team meeting, case conference) ____

Sourcing specialised equipment and resources

Administrative tasks - ACFI

0 10 20 30 40 50 60 70 80 90 100

Administrative Tasks - non-ACFI										
Quality Assurance project/meeting and audits										
Other [ ]										
Other [ ]										

Page Break

Q28

What do you estimate is the average duration (minutes) you spend with a resident in a single standard session at your RACF(s)?
(If you do not complete the specified task please place a '0' in the box)

	Minutes Spent
New Admission Assessment	
Discharge session	
ACFI assessment	
Non-ACFI assessment	
Regular scheduled review	
Unscheduled review	
Falls review	
ACFI treatment	
Non-ACFI treatment	
Resident and family liaising	

Import from library Add new question

Add Block

Administration Questions

Q42

What validated outcome measures do you commonly utilise in the RACF setting? (please tick as many options as are relevant, and add any other measures not listed)

- ☐ Abbey Pain Scale
- ☐ PAINAD
- ☐ M-RVBPI
- ☐ 10m walk test
- ☐ 6m walk test
- ☐ 6 minute walk test
- ☐ 2 minute walk test
- ☐ Elderly mobility scale (EMS)
- ☐ Tinetti performance orientated mobility assessment
- ☐ Physical mobility scale (PMS)
- ☐ Timed up and go test
- ☐ Berg balance scale (BBS)
- ☐ (Modified) functional reach test
- ☐ 30 second sit to stand test
- ☐ Five times sit to stand test
- ☐ Barthel index (BI)
- ☐ Functional independence measure (FIM)
- ☐ SF-36
- ☐ EQ-5D
- ☐ Goal attainment scoring (GAS)
- ☐ Other ____________

Q24

What are the referral methods from which your patient-load is acquired? (please tick as many options as are relevant)

- ☐ General Practitioner
- ☐ Nurse
- ☐ Resident
- ☐ Family
- ☐ Routine for new admission
- ☐ Routine for scheduled review (please specify frequency of reviews, e.g. annual, six monthly) ____________
- ☐ Blanket referral for specific incidents/issues (please specify, e.g. falls, ACFI) ____________
- ☐ Other: ____________

Q27

What do you estimate is the average duration you treat a resident for per a condition/issue/referral?
(please select one duration category for both ACFI and non-ACFI referrals)

	ACFI referral	Non-ACFI referral
Single Review	☐	☐
Short Term (<4 week)	☐	☐
Mid Term (4-12 weeks)	☐	☐
Long Term (>12 weeks)	☐	☐

Q23

What type of funding is your work covered by?
(please tick as many options as are relevant)

- ☐ ACFI
- ☐ Covered by RACF
- ☐ Medicare
- ☐ DVA
- ☐ Private Insurance
- ☐ Self-Funded (resident or family/guardian)
- ☐ Other: ____________

Page Break

Q32

What type of physiotherapy input do respite residents receive?
(If the RACFs do not accept respite residents please write N/A in the box below)

____

Q26

If residents are excluded from physiotherapy care, what is the reason?

- ☐ None Excluded
- ☐ Respite status
- ☐ Cognitive Impairment
- ☐ Funding
- ☐ Maximum ACFI claim reached
- ☐ Other: ____________
- ☐ Other: ____________

Import from library

Add new question

Add Block

Barriers and facilitators

Q12

What are barriers to providing best-practice physiotherapy at your workplace?
Please elaborate in the space below.

Q13

What are facilitators/enablers to providing best-practice physiotherapy at your workplace?
Please elaborate in the space below.

Import from library Add new question

Add Block

End of Survey

Thank you for taking the time to complete our survey.

Your responses have been recorded.

Your participation in our study is greatly appreciated, and your responses will make a valuable contribution to understanding how physiotherapy is currently utilised in Australian RACFs.
