# Supplementary material for: The type and scope of physiotherapy is under-utilised in Australian residential aged care facilities: a national, cross-sectional survey of physiotherapists
Source: BMC Geriatr. 2022 Jul 28;22:625. doi: 10.1186/s12877-022-03248-4 (PMC9331124; doi:10.1186/s12877-022-03248-4)
Supplement: Supplementary file 3 — Additional file 3: Supporting Information Table 2 Extension of Table 1. Allied health professionals in RACFs. [file 12877_2022_3248_MOESM3_ESM.docx]

| **Supporting Information Table 2**  **Extension of Table 1: Allied health professionals in RACFs** | |
| --- | --- |
| **Average number of AHPs across RACFs per participant,** median (range) *(9.5% missing data)* | |
| Other physiotherapists | 0.40 (0-5) |
| Occupational therapists | 0.40 (0-5) |
| Podiatrists | 1.0 (0-12) |
| Chiropractors | 0.0 (0-3) |
| Exercise physiologists | 0.0 (0-1) |
| Diversional therapists | 0.0 (0-5) |
| Dieticians | 0.0 (0-2) |
| Speech and language therapists | 0.0 (0-2) |
| Palliative care nurses/consultants | 0.0 (0-15) |
| Psychologists | 0.0 (0-1) |
| Leisure and Recreational/Lifestyle officers | 0.0 (0-5) |
| Allied health assistants | 0.0 (0-11) |
| ACFI-only allied health professionals |  |
| *ACFI-only Occupational therapists* | 0.0 (0-25) |
| *ACFI-only Podiatrists* | 0.0 (0-5) |
| *ACFI-only Chiropractors* | 0.0 (0-1) |
| *ACFI-only Osteopaths* | 0.0 (0-0) |
