# Supplementary material for: The type and scope of physiotherapy is under-utilised in Australian residential aged care facilities: a national, cross-sectional survey of physiotherapists
Source: BMC Geriatr. 2022 Jul 28;22:625. doi: 10.1186/s12877-022-03248-4 (PMC9331124; doi:10.1186/s12877-022-03248-4)
Supplement: Supplementary file 4 — Additional file 4: Supporting Information Table 3 Extension of Table 2. Detailed interventions and outcome measures reported. [file 12877_2022_3248_MOESM4_ESM.docx]

| **Supporting Information Table 3**  **Extension of Table 2: Detailed interventions and outcome measures reported** | |
| --- | --- |
| **Type of respiratory interventions** |  |
| Education | 7/87 (8.0) |
| Breathing exercises | 67/87 (77.0) |
| Positioning/mobilisation | 26/87 (29.9) |
| Manual techniques | 29/87 (33.3) |
| Provision of equipment | 27/87 (31.0) |
| Unspecified | 9/87 (10.3) |
| **Type of electrotherapy used** |  |
| TENS | 40/65 (61.5) |
| Laser | 5/65 (7.7) |
| TENS and ultrasound | 5/65 (7.7) |
| TENS and laser | 5/65 (7.7) |
| Other (wax bath; electronic massager; TENS and wax bath; TENS and electronic massager) | 10/65 (15.4) |
| **Type of equipment prescriptions** |  |
| Mobility aids | 93/110 (84.5) |
| Manual handling | 29/110 (26.4) |
| Functional aids | 48/110 (43.6) |
| Pressure care | 25/110 (22.7) |
| Personal aids | 25/110 (22.7) |
| Falls risk minimisation | 16/110 (14.5) |
| Exercise | 11/110 (10.0) |
| Unspecified | 6/110 (5.5) |
| **Type of exercise prescribed** |  |
| Balance | 151/169 (89.3) |
| Strength | 156/169 (92.3) |
| Maintenance of range of movement/motion | 156/169 (92.3) |
| Endurance | 79/169 (46.7) |
| Walking and gait re-education | 151/169 (89.3) |
| Functional | 131/169 (77.5) |
| Tai Chi | 19/169 (11.2) |
| Dance | 23/169 (13.6) |
| Pilates | 11/169 (6.5) |
| Hydrotherapy | 9/169 (5.3) |
| Vestibular | 18/169 (10.7) |
| Pelvic floor muscle training | 24/169 (14.2) |
| Other^a^ | < 5 |
| **Regions for joint mobilisation** |  |
| Spinal | 6/35 (17.1) |
| Unspecified or various | 26/35 (74.3) |
| Other (peripheral; traction) | < 5 |
| **Outcome measures used** |  |
| Abbey Pain Scale (APS) | 154/175 (88.0) |
| Pain Assessment in Advanced Dementia Scale (PAINAD) | 42/175 (24.0) |
| Modified Resident's Verbal Brief Pain Inventory (M-RVBPI) | 64/175 (36.6) |
| 10-metre walk test | 39/175 (22.3) |
| 6-metre walk test | 19/175 (10.9) |
| 6-minute walk test | 20/175 (11.4) |
| 4-metre walk test | < 5 |
| 2-minute walk test | 11/175 (6.3) |
| Elderly Mobility Scale (EMS) | 15/175 (6.8) |
| Tinetti Performance Oriented Mobility Assessment (POMA) | 6/175 (3.4) |
| Physical Mobility Scale (PMS) | 99/175 (56.6) |
| Timed-Up-And-Go (TUG) Test | 102/175 (58.3) |
| Berg Balance Scale (BBS) | 72/175 (41.1) |
| Modified Functional Reach (mFR) Test | 27/175 (15.4) |
| 30-second Sit-to-Stand (STS) Test | 27/175 (15.4) |
| 5-times Sit-to-Stand (STS) Test | 63/175 (36.0) |
| Barthel Index (BI) | 8/175 (4.6) |
| Functional Independence Measure (FIM) | 7/175 (4.0) |
| 36-Item Short Form Survey (SF-36) | < 5 |
| European Quality of Life Five Dimension (EQ-5D) | < 5 |
| Goal Attainment Scale (GAS) | < 5 |
| Visual Analogue Scale (VAS) - Pain | 8/175 (3.6) |
| Modified Dynamic Gait Index (mDGI) | < 5 |
| Numeric Rating Scale - Pain | < 5 |
| Clinical Test of Sensory Interaction on Balance | < 5 |
| de Morton Mobility Index (DEMMI) | < 5 |
| Falls Risk Assessment Tool (FRAT) | < 5 |
| 9-hole peg test | < 5 |
| Patient Specific Functional Scale (PSFS) | < 5 |
| 4-Stage Balance Test | < 5 |

^a^Other included: Body awareness; Multiple: motor relearning, dual task, Parkinson’s disease specific, dementia specific
